# Relation between Temperament and Body Mass Index in Teenage Girls

**Published:** 2018-12

**Authors:** Akram GHANBARI MOGHADDAM, Masoomeh ANDISH, Hanieh HEMATI, Rahim AKRAMI, Mojtaba MOHAMMADI, Fereshteh GHORAT

**Affiliations:** 1. Iranian Research Center on Healthy Aging, Sabzevar University of Medical Sciences, Sabzevar, Iran; 2. Student Research Committee, Sabzevar University of Medical Sciences, Sabzevar, Iran; 3. Traditional and Complementary Medicine Research Center, School of Medicine, Sabzevar University of Medical Sciences, Sabzevar, Iran

## Dear Editor-in-Chief

In recent years, the prevalence of obesity in children and adults has been increasing around the world. Various factors such as genetic, environmental and physiological factors can be effective in determinate the Body Mass Index (BMI). These factors affect the absorption and removal of energy in the body by the effect of physiological mediates (1).

Temperament (Mezaj) is a construct of geneticsand environmental style. Based on Persian medicine, temperament includes two main subscales consist of “warm or cold” and “moist and dry” and combination of them.Temperament is a potential factor determining individual differences in weight gain (2, 3).

The resent study examines associations between temperament and change in BMI (4). Temperament, as assessed by a nine-item temperament questionnaire, was related to body composition and non-resting energy expenditure (NREE) (5). In addition, a prospective cohort study demonstrated association between temperament and BMI in adult men and women (6). However, withour search there is not any the same study in temperament of adolescents and BMI. The objectives of these study were to assess whether temperament is associated with BMI in teenager girls.

This cross-sectional study was performed during Oct 2015 to Jul 2016, on 360 girls’ of high school students in Sabzevar, northeast of Iran.

The study was approved by the Ethics Committee of Sabzevar University of Medical Sciences (IR.MEDSAB.REC.1394.130).

The study samples were selected in a systematic cluster. Data were collected by a questionnaire containing two demographic sections and a standard temperament questionnaire (7). Data were analyzed using descriptive statistics and chi-square statistical analysis using SPSS ver. 21 (Chicago, IL, USA). The *P*≤0.05 wasconsidered statistically significant.

The finding showed that mean age of girls was 16 ± 1 yr. The 38% of girls had BMI≤ 18.5%, 12.3% overweight (BMI≥25) and 1.7% obesity (BMI ≥30). Results due toBMI and type of temperament are shown in Table 1. There was not a significant relationship between hot/cold temperament and BMI. However, there was a significant difference between the BMI and the dry or moist temperament. In other words, the highest percentage of girls with BMI≤18 had dry temperament and the highest percentage of overweight and obese girls had moist temperate. In addition, the findings of ANOVA test showed that the type of combined temperament was closely related to height, weight, and BMI, and this relationship is statistically significant (*P*<0.001) (Table 2). Other finding showed that there was a significant relationship between dry temperaments with low BMI. Overweight and obesity were observed in moist temperament.

**Table 1:** Relation between BMI and type of single and combined temperament in teenage girls

***Variable***	***BMI<18.5 N(%)***	***18.5<BMI<24.9 N(%)***	***25<BMI<29.9 N(%)***	***BMI>30 N(%)***	***Qui-square Test***
Single Temperament					
Cold	34(9.4)	53(14.7)	11(3)	0	*P*=0.106
Hot	18(5)	41(11.5)	17(4.7)	2(0.5)	
Moderate	48(13.3)	112(31.1)	19(5.4)	5(1.4)	
Single Temperament					
Dry	49(13.6)	41(11.4)	4(1.1)	0	*P*<0.001
Moist	8(2.2)	74(20.5)	30(8.4)	5(1.4)	
Moderate	43(11.9)	91(25.3)	13(3.7)	2(0.5)	
Combination Temperament					
Cold & Moist	12(3.3)	59(16.4)	17(4.7)	0	*P*<0.001
Cold & Dry	45(12.5)	29(8)	6(1.7)	3(0.8)	
Hot & Moist	15(4.2)	76(21.2)	22(6.1)	4(1.1)	
Hot & Dry	28(7.8)	42(11.7)	2(0.5)	0	

**Table 2:** Relation of combined temperamental type with BMI, height and weight in teenage girls

***Variable*** *(Mean±SD)*	***Hot & Moist***	***Cold & Moist***	***Hot & Dry***	***Cold & Dry***	***ANOVA Test***
BMI	24.1±3.7	21.7±3.5	19.3±4.1	18.6±2.5	*P*< 0.001
Hight (cm)	160±6.2	161±6.5	162±5.9	156±5.9	*P*< 0.001
Weight (kg)	63±1.2	56±7.8	51±1.1	45±6.7	*P*< 0.001

The results are consistent with another study (5). In this study, the relationship was reported with physical inactivity and overweight. Moisture and cold cause deposition of more fat in cells and accumulation of fat in certain areas of the body (8). However, the present study did not show a relationship between cold temperament and increase of BMI. Contrary to other studies, higher body mass was found to be significantly higher in hot and moist temperament. There was a significant correlation between dry temperament and low BMI.

Possible mechanisms of the effect of temperate on body mass can be the activation of nervous system, increased thermosensitivity, the expression of various genes, and fat oxidation (6).

As a conclusion, temperament is a combination of interactions between genetic and environmental factors and one of important factors that determinate range of BMI for an individual. Our finding give support for role of temperament in weight gain. Knowledge of temperament can be used for motivating weight loss and development weight management interventions. However, it is necessary to conduct more comprehensive studies in this field to confirm the finding of this result.
